# Repeated methylene blue administration produces analgesia in experimental pain

**DOI:** 10.1186/1129-2377-14-S1-P86

**Published:** 2013-02-21

**Authors:** A Dondas, A Luca, T Alexa, VA Grigoras, OC Mungiu, CR Bohotin

**Affiliations:** 1 Centre for the Study and Therapy of Pain, University of Medicine and Pharmacy, Grigore. T. Popa, Romania; 2 Department of Neurology, Rehabilitation Hospital, University of Medicine and Pharmacy, Grigore. T. Popa, Romania

## Introduction

Methylene blue (MB), a widely used inhibitor of NO activity/production, is also a reduction-oxidation agent that can act both as a powerful antioxidant and as an enhancer of the electron transport chain. Furthermore, it prevents formation of mitochondrial oxygen free radicals and promotes oxygen consumption ( Atamna et al. 2010 , Rojas et al. 2012 ).

## Purpose

The aim of the study was to investigate the effects of chronic (14 days) MB administration on experimental pain in mice.

## Methods

Sixteen Swiss male mice were divided into 2 groups: control group (n=8) and MB group (n=8); both groups received daily injections, for 14 days, either with saline 20 Î¼l i.p. (control group) or with MB 5 mg/kg b.w. i.p (MB group). Nociceptive tests (tail flick and hot plate) as well as mechanical and thermal withdrawal thresholds were measured every two days before MB/ saline administration. Before and two hours after the last dose was administered (day 14), each group was evaluated for the nociceptive tests and heat/mechanical hyperalgesia; results were compared with paired Student’s t test. After nociceptive tests, the mice received 20 Î¼l of 5% formalin into the upper right lip and the intensity of the orofacial pain was assessed. The results were compared with those from saline group using unpaired Student’s t test.

## Results

Chronic administration of MB increased tail flick and hot plate latencies (p=0.03, p=0.02). We also noted an increase in the reaction time for thermal hyperalgesia assessed by Hargreaves method (p=0.04). As for the formalin-induced orofacial pain, MB produced a significant analgesic effect on both phases (p=0.03).

## Conclusion

Our study demonstrates that chronic administration of MB has analgesic effects on acute nociception as well as on the orofacial inflammatory pain; further studies must be conducted in order to elucidate the mechanism by which the methylene blue exerts its antinociceptive effect.
